# The compensatory hypertrophy of transferred flexor hallucis longus tendon for insertional Achilles tendinopathy: a retrospective MRI study

**DOI:** 10.1038/s41598-023-47725-1

**Published:** 2023-11-22

**Authors:** Wei-Jen Liao, Shih-Chieh Tang, Han-Ting Shih, Kuo-Chih Su, Kao-Chang Tu, Shun-Ping Wang

**Affiliations:** 1 Department of Orthopaedics, Taichung Veterans General Hospital, Taichung, 407 Taiwan; 2 Department of Medical Research, Taichung Veterans General Hospital, Taichung, Taiwan; 3 Department of Biomedical Engineering, Hung Kuang University, Taichung, 433 Taiwan; 4 Department of Post‐Baccalaureate Medicine, College of Medicine, National Chung Hsing University, Taichung, 40227 Taiwan

**Keywords:** Musculoskeletal system, Medical imaging, Therapeutics

## Abstract

Flexor hallucis longus (FHL) transfer is an effective surgery in treating insertional Achilles tendinopathy (IAT). However, limited data exist regarding the post-surgery changes in the transferred FHL. The study aimed to compare the sequential changes and hypertrophy of FHL after isolated FHL transfer (FHLT). We retrospectively enrolled patients who underwent isolated FHLT for insertional Achilles pathology from 2015 to 2020 and divided them into two groups based on whether reattachment of the residue Achilles stump to the FHL was performed or not. We recorded demographic characteristics, MRI parameters, and functional outcome. We also analyzed the correlation between the collected data and FHL hypertrophy. Results revealed no significant differences in most MRI parameters of FHL and functional outcomes between the groups. However, the fat distribution within the FHL showed significant reduction and notable 20.2% hypertrophy after FHLT. Interestingly, the hypertrophy of the FHL was significantly more pronounced in the non-reattached group. Furthermore, we observed a positive correlation between the follow-up period and FHL hypertrophy. In conclusion, the FHL demonstrated significant enlargement over time following FHLT. The compensatory hypertrophy of the transferred FHL was particularly evident and the cumulative incidences of FHL enlargement over time were higher in the non-reattached groupcompared to reattached group. However, both reattachment and non-reattachment of Achilles stump on FHL transfer for insertional Achilles tendinopathy carried similar postoperative functional outcomes.

## Introduction

The Achilles tendon plays a key role in facilitating daily activities^1^. Insertional Achilles tendinopathy (IAT) can lead to chronic pain or disability and may compromise quality of life. Overuse, poor blood supply, genetic factors, and inflammation and infection are the most commonly reported causes^2–4^. Conservative treatments achieve successful outcomes in 65% to 90% of cases of IAT^5^. If a conservative treatment fails, surgical intervention can be an effective approach for treating IAT. In low-grade IAT, partial debridement of the Achilles insertion site followed by reconstruction with a suture anchor was an effective treatment^6^. For severe cases with irreparable IAT lesions, chronic rupture of the distal Achilles tendon, or loss of Achilles insertion due to debridement surgery for infection, tendon transfer is a surgical consideration. The hamstring, soleus peroneus brevis, flexor digitorum longus, and flexor hallucis longus (FHL) are candidates for tendon transfer in IAT^7–10^.

FHL transfer (FHLT) has been proposed as an effective surgical method for treating Achilles pathology^9–11^. FHLT can be categorized into two types: FHL augmentation, which addresses the Achilles defect while preserving the original Achilles insertion on the calcaneus; and isolated FHLT, which involves replacing the diseased Achilles entirely and excising the distal Achilles along with its insertion. FHL augmentation is indicated for chronic or neglected Achilles ruptures with a short gap and a relatively healthy insertion^12–14^. Severe IAT which required surgical intervention should consider FHL transfer to enhance plantarflexion strength^15,16^. Compared to single skin incision, double skin incision could harvest longer tendon graft by medial incision near the knot of Henry. However, Isolated FHLT through both single and double incisions have been proposed as an effective surgery with positive outcomes for severe IAT^17–19^.

FHL augmentation has been extensively discussed and proposed to remedy Achilles defects and increase the load to failure^20^. Although most surgically treated patients experienced partial deficits in plantarflexion strength^13,14^, FHL augmentation has demonstrated efficacy in restoring gait function and enabling resumption of daily activities^19,21,22^. Oksanen et al. and Hahn et al. have reported an average hypertrophy of 52% in the FHL muscle and a 17% increase in FHL volume of muscle belly, respectively, based on the follow-up magnetic resonance imaging (MRI) after FHL augmentation^13,14^. However, their studies compared the non-operated site of FHL and their methods of FHL measurements lacked detailed descriptions in their MRI studies. Moreover, changes in FHL muscle size following isolated FHLT, as opposed to FHL augmentation, have not been previously reported.

**Figure 1:**
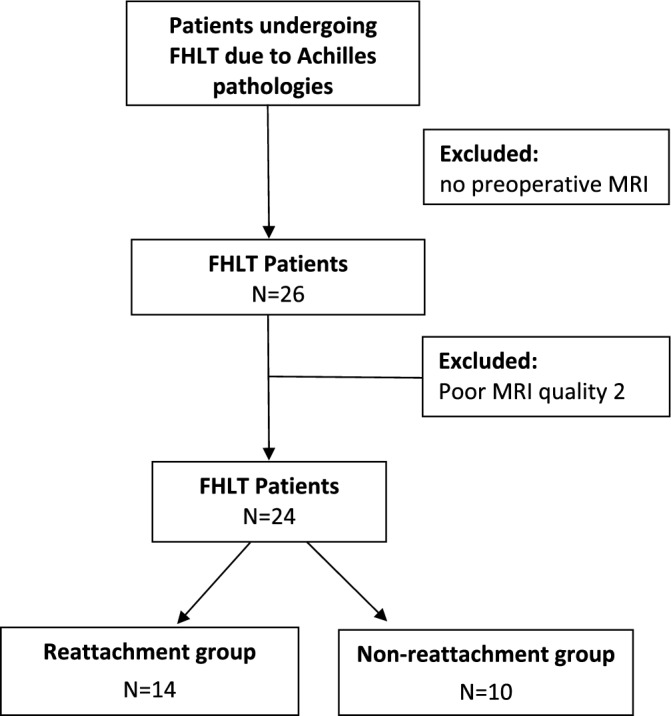
Flowchart of the enrolled participants.

According to the condition of Achilles stump after adequate debridement, the possibility of reattachment would be evaluated during surgery. Two management approaches arise: reattachment or non-reattachment of the Achilles stump to the transferred FHL (Fig. 1). Various outcomes following reattachment and non-reattachment of the residual Achilles to transferred FHL have been reported in cases of severe IAT^17–19,21,22^. The belly of the FHL tendon was sutured to the Achilles stumps was described by Wilcox et al. and Mahajan et al.^21,22^. However, a comprehensive investigation into the changes of the transferred FHL in IAT concerning reattachment or not has not been undertaken. The study aimed to compare the sequential changes and hypertrophy of FHL after isolated FHLT with various management of Achilles stump. We hypothesize that the muscle belly of the transferred FHL would exhibit hypertrophy, and the fat distribution would decrease in non-reattachment group after surgery.

## Results

### Demographics

We retrospectively enrolled 28 patients who underwent isolated FHLT. Two patients were excluded due to poor quality MRI scans obtained from other hospitals, and an additional two patients were excluded as they did not have preoperative MRI studies performed. Among the remaining 24 patients included in the study, 14 were categorized within the reattachment group and the remaining 10 were assigned to the non-reattachment group. (Fig. 1) The two groups displayed no significant differences in terms of age, gender, BMI, affected side, ASA, tendinopathy etiology, or follow-up duration (Table 1).

**Table 1:** Characteristics of enrolled patients.

	Reattachment	Non-reattachment	*p* value
Total patients (n = 24)	14 (58.3%)	10 (41.7%)	
Gender			0.272
Female	13 (92.9%)	7 (70.0%)	
Male	1 (7.1%)	3 (30.0%)	
Mean age (years)			
Mean ± SD	60.0 ± 9.1	63.9 ± 10.3	0.337^†^
Median (range)	62.0 (38.0–70.0)	66.00 (40.0–76.0)	0.240^†^
BMI (kg/m^2^)			
Mean ± SD	26.8 ± 3.8	26.1 ± 3.3	0.621^†^
Median (range)	27.2 (19.6–33.2)	25.2 (22.2–31.1)	0.520^†^
ASA grade			
Mean ± SD	1.9 ± 0.27	2.2 ± 0.42	0.066
Median (range)	2.0 (2.0–2.0)	2.0 (2.0–2.3)	0.067
Side			0.678
Left	6 (42.9%)	3 (30.0%)	
Right	8 (57.1%)	7 (70.0%)	
Mechanism			0.597
Rupture	11 (78.6%)	6 (60.0%)	
Infection	2 (14.3%)	3 (30.0%)	
Tendinopathy	1 (7.1%)	1 (10.0%)	
Follow-up period (months)			
Mean ± SD	37.14 ± 16.78	24.90 ± 10.52	0.054^†^
Median (range)	34.50 (9.0–64.0)	24.50 (10.0–44.0)	0.069^†^
Bone tunnel			
Distance (mm)	6.9 (3.1–10.0)	7.3 (2.2–10.5)	0.792^†^
Angle (lateral view)	79.5 (58.0–89.0)	84.0 (69.8–87.0)	0.411^†^

Chi-square test. ^†^Mann–Whitney *U* test or independent t test.

### Parameters of postoperative MRI between groups

We found no significant differences between the two groups in terms of postoperative hypervascularization in T2-weighted images, homogeneity in T1-weighted images, integration at the reattachment site, FHL union rates at the tunnel site, or fatty infiltration evaluated using Goutallier, Fuchs classification systems, as well as software calculation. In both the reattachment and non-reattachment groups, the bone tunnel distances were 6.9 and 7.3 mm (p = 0.792), respectively, while the tunnel angles in the sagittal plane were 79.5° and 84° (p = 0.411), respectively. These groups did not exhibit significant differences in these parameters (Table 2).

**Table 2:** Comparative parameters in postoperative MRI.

	Reattachment	Non-reattachment	*p* value
Inflammation (T2W)	0 (0.0%)	0 (0.0%)	
Homogeneity (FHL, T1W)	12 (85.7%)	9 (90.0%)	1.000
Integration at reattached site	14 (100.0%)	–	–
FHL union in bone tunnel	13 (92.9%)	10 (100.0%)	1.000
Goutallier grade			0.952
Grade 0	4 (28.6%)	3 (30.0%)	
Grade 1	8 (57.1%)	6 (60.0%)	
Grade 2	2 (14.3%)	1 (10.0%)	
Grade 3	0 (0%)	0 (0%)	
Grade 4	0 (0%)	0 (0%)	
Fuchs stage			1.000
Minimal	12 (85.7%)	9 (90.0%)	
Moderate	2 (14.3%)	1 (10.0%)	
Severe	0 (0%)	0 (0%)	
% Fatty infiltration	2.55 (1.19–3.52)	3.36 (1.39–5.79)	0.412^†^

*T1W* T1-weighted, *T2W* T2-weighted.

Chi-square test. ^†^Mann–Whitney *U* test, median (range).

### Comparison of preoperative and postoperative FHL hypertrophy

The median preoperative and postoperative FHL areas at the selected level increased significantly by 20.2% in the entire FHLT cohort (Table 3). The subgroup analysis results indicated that the FHL area increased by 30.5% after surgery in the reattachment group; however, this finding did not reach statistical significance (*p* = 0.124). In the non-reattachment group, the cross-sectional area of the FHL muscle increased significantly after surgery (*p* = 0.017). Furthermore, postoperative fatty distribution calculated by Mimics Innovation Suite software in the FHL decreased significantly in both groups.

**Table 3:** Comparison of pre- and postoperative fat infiltration and FHL area.

	Pre-op	Post-op	*p* value
Reattachment			
Fat (%)	14.41 (12.81–23.83)	2.55 (1.19–3.52)	0.016*
FHL area (mm^2^)	491.51 (359.04–1080.82)	641.85 (353.22–930.86)	0.124
Non-reattachment			
Fat (%)	18.32 (11.56–35.42)	3.36 (1.39–5.79)	0.005**
FHL area (mm^2^)	563.24 (198.44–699.32)	637.30 (480.78–1183.40)	0.017*
Total			
Fat (%)	15.48 (12.88–30.77)	2.79 (1.29–4.46)	< 0.001**
FHL area (mm^2^)	529.78 (198.44–1080.82)	637.30 (353.22–1183.40)	0.009**

Wilcoxon signed-rank test; *p < 0.05, **p < 0.01.

### Clinical outcomes and complications after FHLT surgery

The median AOFAS hindfoot scores increased significantly from 61.5 to 100 in the reattachment group and from 65 to 100 in the non-reattachment groups. The VAS scores decreased significantly after FHLT surgery. However, the subgroup analysis results revealed no significant differences in preoperative and postoperative AOFAS or VAS changes between the reattachment and non-reattachment groups (p = 0.837). One patient in the reattachment group sustained a minor wound complication with delayed wound healing with superficial infection, but it healed uneventfully after local wound care and antibiotic treatment. The functional outcome was still good in this case. No significant difference in complication rate was observed between the groups (Table 4).

**Table 4:** Outcomes after FHL transfer.

	Reattachment	Non-reattachment	*p* value
AOFAS			
Pre-op	61.50 (40.00, 69.00)	65.00 (32.00, 70.00)	0.656^†^
Post-op	100.00 (77.00, 100.00)	100.00 (87.00, 100.00)	0.672^†^
Δ AOFAS	38.00 (31.00, 57.00)	35.00 (25.00, 60.00)	0.837^†^
VAS			
Pre-op	7.50 (5.00, 10.00)	7.00 (5.00, 8.00)	0.163^†^
Post-op	0.00 (0.00, 1.00)	0.00 (0.00, 2.00)	0.333^†^
Complication	1 (7.1%)	0 (0.0%)	1.000

Chi-square test. ^†^Mann–Whitney U test.

### Factors correlated with postoperative change in FHL area

The correlation analysis revealed no significant correlations of age, BMI, clinical outcome, tunnel distance, tunnel angle, ASA grade, or preoperative FHL area and postoperative FHL area. Follow-up duration was the only factor that was significantly correlated with the postoperative change in the FHL area (p = 0.038) (Table 5).

**Table 5:** Coefficients of correlation with ΔFHL area.

	*r*	*p* value
Age	− 0.200	0.349
BMI	− 0.119	0.579
Δ AOFAS	0.073	0.736
Tunnel distance	0.019	0.929
Tunnel angle (LAT)	− 0.027	0.902
ASA grade	− 0.319	0.129
Follow up period (day)	0.425	0.038*

Spearman's rho coefficient. *p < 0.05.

### Cumulative incidence of FHL enlargement over time in two groups

**Figure 2:**
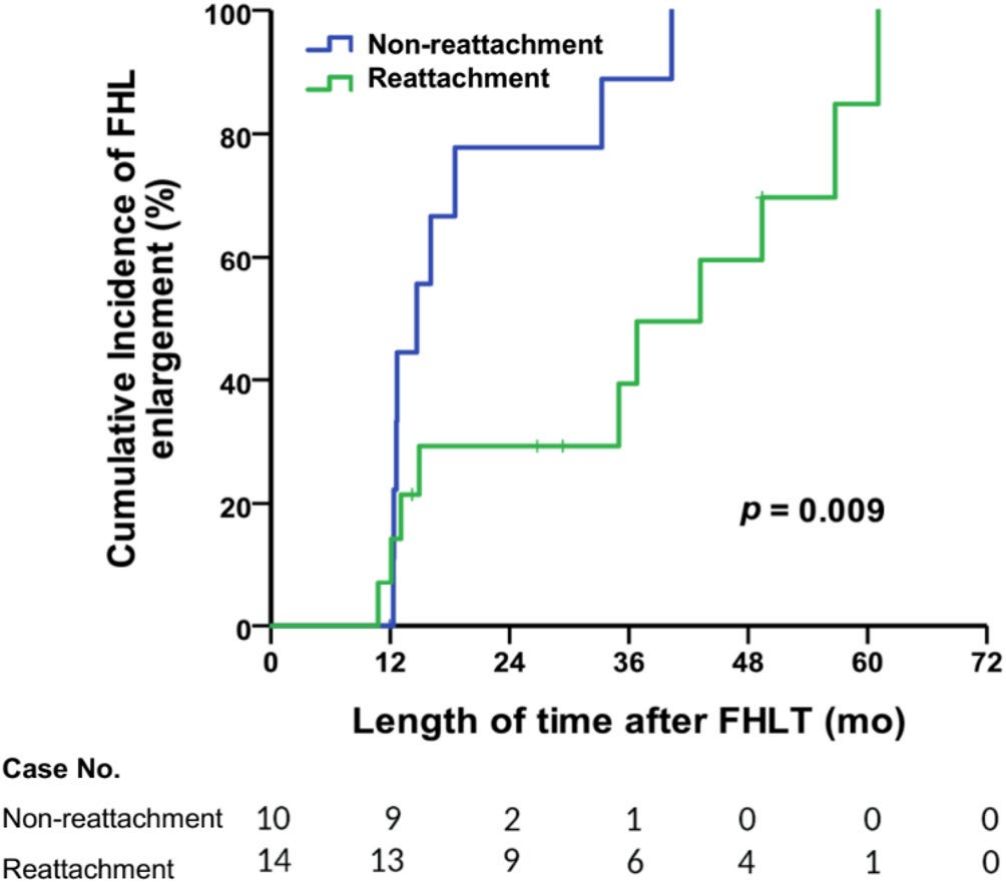
The cumulative incidences of FHL enlargement (%) over time after FHLT in the reattachment and non-reattachment groups were analyzed using Kaplan–Meier method.

The FHL enlargement over time was further analyzed using both the Kaplan–Meier method and the log-rank test. The significant differences (*p* = 0.009) between two groups on cumulative incidence of FHL enlargement were identified since 18 months after FHLT. In Fig. 2, The y-axis means cumulative incidence of FHL enlargement (%) and the x-axis means time duration after FHLT surgery.

## Discussion

IAT can cause chronic pain or rupture, resulting in considerable impairment of the patient’s everyday life. For patients with severe IAT lesions, isolated FHLT through a single incision is an effective surgical option. The transferred FHL will experience significant strain after the resection of the distal part of the diseased Achilles tendon, which includes its insertion. The dominant role of the new tendon rather than triceps surae muscle and its alterations after surgery were of interest to us and should be further investigated. After FHLT, the VAS scores in the reattachment and non-reattachment groups were significantly reduced, and scores of functional outcome indicators increased. Complication rates were also relatively low. We thus argue that FHLT, both with and without Achilles reattachment, is a safe and effective procedure for treating chronic IAT or tear in the distal part of the FHL. There were no significant differences in the measured parameters of FHL on MRI between the reattachment and non-reattachment groups. The results of this MRI study demonstrate that the percentage of fat distribution was significantly reduced and the FHL muscle exhibited 20.2% hypertrophy after FHLT. Furthermore, the enlargement of the cross-section area after FHLT was significantly correlated with duration of follow-up. These findings support our hypothesis in this study.

The AOFAS score and the VAS pain scale were used to evaluate the functional outcome. Since the findings of functional outcomes are not significant between two groups. In this current study, we dedicate ourselves to exploring and comparing various possible factors influencing the changes in the transferred FHL tendon, including complication, inflammation, fat degeneration, homogeneity determined by the Goutallier and Fuchs classification system, integration of the tendon to bone and the position of bone tunnel (demonstrated in Table 2). The transferred FHL on MRI of all patients healed well and was devoid of degenerative signs, dysfunction, or FHL rupture. Besides, there are no significant differences in MRI parameters except the enlargement and the fat distribution of FHL. We assume that the differences in the enlargement and the fat degeneration of FHL in the two groups may not play a dominant role in the functional outcome. One possibility of a similar outcome between subgroups is that the scoring systems may not have enough sensitivity to differentiate the subtle differences in these cases. However, all patients returned their daily activities consisting of level walking, stair climbing, and pre-injured activities during follow-up visits in these two subgroups. To date, it remains unclear whether reattachment of the Achilles residue to the FHL has a superior outcome, and we need more high-level research to elucidate this issue.

Regarding the hypertrophy of the cross-sectional area of FHL in the subgroup analysis, the FHL area significantly increased in the non-reattachment group but not in the reattachment group. This implies that the strength of the initial isolated FHLT without Achilles reattachment was not sufficient to completely replace the plantarflexion force of the Achilles in the early post-operative stages after the operation. A greater tendon–muscle complex strength and size are required to replace the original Achilles function. This finding is consistent with results of previous studies that reported a 24–36% deficiency in ankle plantarflexion strength after FHLT while it was compared with the non-operated side^13,23,24^. We also observed that the transferred FHL became larger with time and significant enlargement of transferred FHL during the follow-up period in the cases of the non-reattached group as Table 3 and Fig. 2. In the reattachment group, the transferred FHL along with the residual Achilles tendon created ankle plantarflexion together; therefore, we supposed the burden on the transferred FHL tendon was reduced with the assistance of the residual Achilles and the absence of additional load caused no significant enlargement of FHL in cases with Achilles reattachment. Reattaching the residual Achilles to the transferred FHL may be a favorable choice for improving the postoperative power of ankle plantarflexion. However, further studies are required to confirm this.

Several types of tendon transfer and augmentation can be used to treat Achilles defects or pathologies. However, few studies have examined postoperative changes in the grafted tendon on MRI scans. The MRI parameters and number of cases examined in most studies are quite limited, and none of these studies have thoroughly explained their methods of measuring the area of the transferred tendon^13,14^. Li et al. evaluated the changes in an autograft hamstring tendon after Achilles reconstruction and reported that the grafted tendon offered satisfactory tension and continuity to compensate for the absence of an Achilles tendon^25^. However, they did not evaluate other parameters in their MRI study.

According to our analysis results, the cross-sectional area of the FHL muscle belly at the same level measured on MRI significantly increased by 20.2% in the entire FHLT cohort (p = 0.009). Our correlation analysis revealed that the amount of FHL hypertrophy was positively correlated with the follow-up period (p = 0.038), i.e., duration of use. This correlation may be due to the compensatory mechanism of the transferred FHL after Achilles dysfunction. The non-reattachment group, even if MRI scans were conducted later in time, exhibits greater hypertrophy, thus indicates that the transferred FHL muscle seems to physiologically compensate for the force of the missing gastrocnemius-Achilles-tendon complex. However, functionally, there were no differences between either the reattachment or non-reattachment of the Achilles stump to the FHL. No other previous studies have proposed that the transferred FHL muscle mass gradually increases over time after FHLT. Nevertheless, Stephan et al. conducted an MRI study and suggested that FHL hypertrophy in patients with functional deterioration of Achilles tendinopathy is a possible compensatory mechanism^26^. Their report on the compensation of FHL accompanying load changes also partially supports our findings. In a case series, Hahn et al. reported that the FHL volume increased by 17%, on average, in patients who sustained chronic Achilles pathologies with intact Achilles insertion and were treated with FHL augmentation^14^. Their report is consistent with our findings. However, the patients they reviewed underwent FHL augmentation, which is not identical to FHLT after total resection of the Achilles insertion. Moreover, they compared the FHL volume on the operated side with that on the non-operated side. Such a comparison may be affected by the greater size of the dominant limb.

There is a paucity of data in the literature on FHL changes in size on MRI after transfer. Oksanen et al. reported that the FHL area increased by 52% after FHLT compared with that on the non-operated side^17^. Their result showed a much larger increase than that observed in a prior study and is also inconsistent with our findings^14^. However, they included only seven patients in their study, and it is known that enrollment of very limited cases tends to result in inaccurate findings. Furthermore, they measured the FHL cross-sectional area at the same distance from the talocrural joint (ankle) before and after the operation; this measurement method might be problematic because it does not con-sider the change in FHL position after FHLT, which pulls the entire muscle belly of the FHL downward. When the cross-sectional area is measured at the same distance from the ankle joint preoperatively and postoperatively, a different cross-sectional area of the transferred FHL will be measured. At the same fixed distance from a specific bony landmark, the measured cross-sectional area of the transferred FHL muscle would be considerably larger than that of the FHL muscle on the non-operated side because the diameter in the proximal part of muscle belly is larger than that in the distal part. The measurement method used in our study avoided these errors. We used the myotendinous junction of the FHL muscle as a reference point and measured the cross-sectional area of the FHL at 80 mm beyond the myotendinous junction. Furthermore, we compared the changes in FHL cross-sectional area on the same side before and after the operation to elucidate the postoperative changes in the same muscle.

In addition to the increase in FHL muscle cross-sectional area, fatty infiltration on MRI decreased significantly from 15.48 (12.88–30.77) to 2.79 (1.29–4.46) after FHLT (p < 0.001). No significant difference in fatty infiltration was observed between the two groups preoperatively or postoperatively. We speculate that the transferred FHL tendon gradually assumes the role of the Achilles tendon, gradually taking over its functions. Through strengthening, both the volume and composition of the transferred FHL muscle undergo changes. The phenomenon of muscle texture changing due to disease or training has been reported in previous studies in other fields^27^. However, there were no significant differences in fat distribution of FHL before and after surgery when evaluated by the Goutallier or Fuchs classification. (data not shown in Table 3) These two grading systems were originally designed for evaluating changes of fat distribution in rotator cuff tear and can effectively grade disease rotator cuff. We postulate that both grading systems are insufficiently sensitive to differentiate the changes of fat infiltration in FHL.

This study has several limitations. First, relatively small number of patients undergoing FHLT for IAT were enrolled in this study. However, our study included 24 participants, a sample size that is larger than the sample sizes of previous studies. Second, the retrospective design of this study is subject to inherent biases, including selection bias. Third, the differences observed in our MRI study may not necessarily be correlated with clinical differences in prognosis or muscle strength. Furthermore, there was obviously varied follow-up-time between the two groups although the difference did not reach the statically significant. This issue might partially impact the confidence to interpret the finding of this study. In the future, further analysis may be required to elucidate the correlation between MRI findings and changes in muscle strength. Despite these limitations, this study corrected the measuring errors in previous studies and provided a detailed measurement method. Furthermore, this study is the first to comprehensively identify and compare changes in FHL muscle parameters on MRI.

## Conclusions

Isolated FHLT through a single incision was reported as an effective surgical treatment for IAT in previous research. We observed a significant increase in the cross-sectional area of the FHL muscle over time on postoperative MRI scans and the cumulative incidences of FHL enlargement during follow-up time after FHLT were higher in non-reattachment group while comparing to reattachment group. Additionally, there was a significant decrease in fatty infiltration after FHLT. The compensatory hypertrophy of the transferred FHL was significantly evident in the non-reattachment group but not in the reattachment group. The results imply that the initial sole transferred FHL without Achilles reattachment gained the inadequate strength for heel plantarflexion after FHLT.

## Materials and methods

### Patient enrollment and groups

This retrospective study was conducted in accordance with the Declaration of Helsinki and all experiments were performed in accordance with guidelines and regulations. Approved by the Institutional Review Board of Taichung Veterans General Hospital (CE21051A) and informed consent has been waived due to the anonymity and retrospective nature of the study. The present retrospective cohort study was conducted at a single medical center. We followed consecutive patients who underwent FHLT for Achilles pathology by single experienced foot and ankle surgeons between 2015 to 2020. Inclusion criteria were as follows: (1) Age more than 18 years old; (2) Received FHLT under the diagnosis of IAT; (3) Follow up period more than 9 months after surgery; (4) Complete clinical assessment before and after surgery. All patients had failed conservative treatment that comprised NSAIDs, rehabilitation, and shoe modification (hindfoot padding) for at least 3 months. All postoperative MRI procedures were performed in our institution after FHLT. The patients were diagnosed as having IAT requiring dissection of more than 50% (in terms of thickness) of the diseased tendon, neglected distal Achilles rupture with diseased insertion, or a post-infection course after failed Achilles reconstruction. Each patient’s diagnosis was confirmed through physical examination and MRI findings. The exclusion criteria were as follows: (1) incomplete or poor-quality MRI study; (2) preoperative and postoperative MRI scans that captured different parts of lower limbs, such as the lower limb and ankle; and (3) concomitant other foot or ankle surgical procedures performed in addition to FHLT.

Patients were divided into two groups according to the management of the distal end of the Achilles stump: (1) reattachment group, in which the FHL was reattached to the resected end of the residual Achilles tendon; and (2) non-reattachment group, in which isolated FHLT occurred without reattachment of the residual Achilles tendon (Fig. 2).The decision regarding reattachment of the residue Achilles depends on the quality of the Achilles stump following thorough debridement. The age, gender, body mass index (BMI), and American Society of Anesthesiologists (ASA) scores of these patients and the diagnoses of Achilles pathologies were recorded. Preoperative and postoperative data included clinical outcomes (American Orthopedic Foot and Ankle Society [AOFAS] and visual analog scale [VAS] scores), radiograph scans, and MRI scans. In order to determine the number of participants needed in this study, we used G*power 3.1.9.7 (Heinrich-Heine-Universität Düsseldorf, Germany) to perform the sample size calculation based on the differences of Achilles hypertrophy before and after FHLT. With an alpha error of 0.05 and power setting at 0.8, the effect size was calculated to be 0.685 using the results according to the previous study^13^, and it was determined that at least 19 participants were needed to achieve sufficient statistical power in this study.

### Surgical intervention and postoperative care

For the operation, each of the patients was placed in the prone position under general anesthesia, and a tourniquet was used. Using the posteromedial approach, the diseased Achilles tendon was recognized and debrided adequately. In all cases, the proximal end of the Achilles stump was retained, while the distal portion of the diseased Achilles tendon, including the insertion footprint on the calcaneus, was completely excised. The deep fascia of the calf was opened. The FHL tendon was identified, and tenotomy was performed distally as far as possible to ensure sufficient donor tendon length. The FHL was cut off at the distal part of the FHL behind the medial malleolus through the same incision^28^. After decorticated the posterior tuberosity of the calcaneus and removed the dorsal heel spur, a bony tunnel over the posterior tuberosity of calcaneus was created using a 5.0-mm cannulated drill bit from top to bottom diagonally. After the FHL tendon passed through the bony tunnel, the tension of the FHL was adjusted with respect to the ankle plantarflexion position. Once the final position was verified, a tenodesis screw (SwiveLocks, 5.5 mm, Arthrex, Naples, USA) was used to fix the transferred FHL. There were two management methods regarding to the Achilles remnant, reattachment or non-attachment to the transferred FHL, in this study. The management depends on the condition of the Achilles remnants after adequate debridement. For Achilles reattachment in FHLT, the proximal Achilles stump was sutured to the muscle belly of the transferred FHL. On the contrary, in the non-reattachment group, the proximal Achilles stump was only left in situ and there were no sutures between the residual Achilles and the transferred FHLT. The surgical wound was irrigated and closed layer by layer. A bivalve splint was applied in a plantarflexion position before the patient regained consciousness.

Postoperative management consisted of the ankle being maintained in plantarflexion for the first 4 weeks with bivalve short-leg splint immobilization. After the splint was removed, partial weight-bearing protection was started with a controlled-ankle-motion walking boot for the next 4 weeks. Active dorsiflexion and plantarflexion were allowed at this period. After 4 weeks with protection provided by the walking boot, patients began passive range-of-motion exercises and stretches to strengthen the ankle. Twelve weeks after the operation, the walking boot was removed, and patients returned to their regular walking and activities.

### MRI examination

All patients received MRI survey before and after surgery. Pre-operative MRI was done within at least 3 months before surgery. Post-operative MRI was performed during 9–12 months after surgery. The target lesions were imaged using a 1.5 Tesla MR scanner (GE Healthcare, Chicago, IL, USA). The MRI scans were performed with a surface coil. The medial malleolus was centered in the coil. The patient kept supine and placed the foot in a relaxed position (plantarflexion: 10°–20° and external rotation: 10°–30°). MRI of ankle in the axial, sagittal, and coronal planes were performed. The axial volume acquisition of 256 × 256 matrices was divided into 1-mm thickness without a gap. The field of view enclosed distal tibia and fibula, ankle joint, tarsal bones, and the bases of the metatarsals. Target localization was performed using T1-weighted (T1W), T2-weighted (T2W), proton density fat saturation, and short tau inversion recovery sequences were obtained without administration of gadolinium.

### Classification and measurement parameters of FHL on MRI

**Figure 3:**
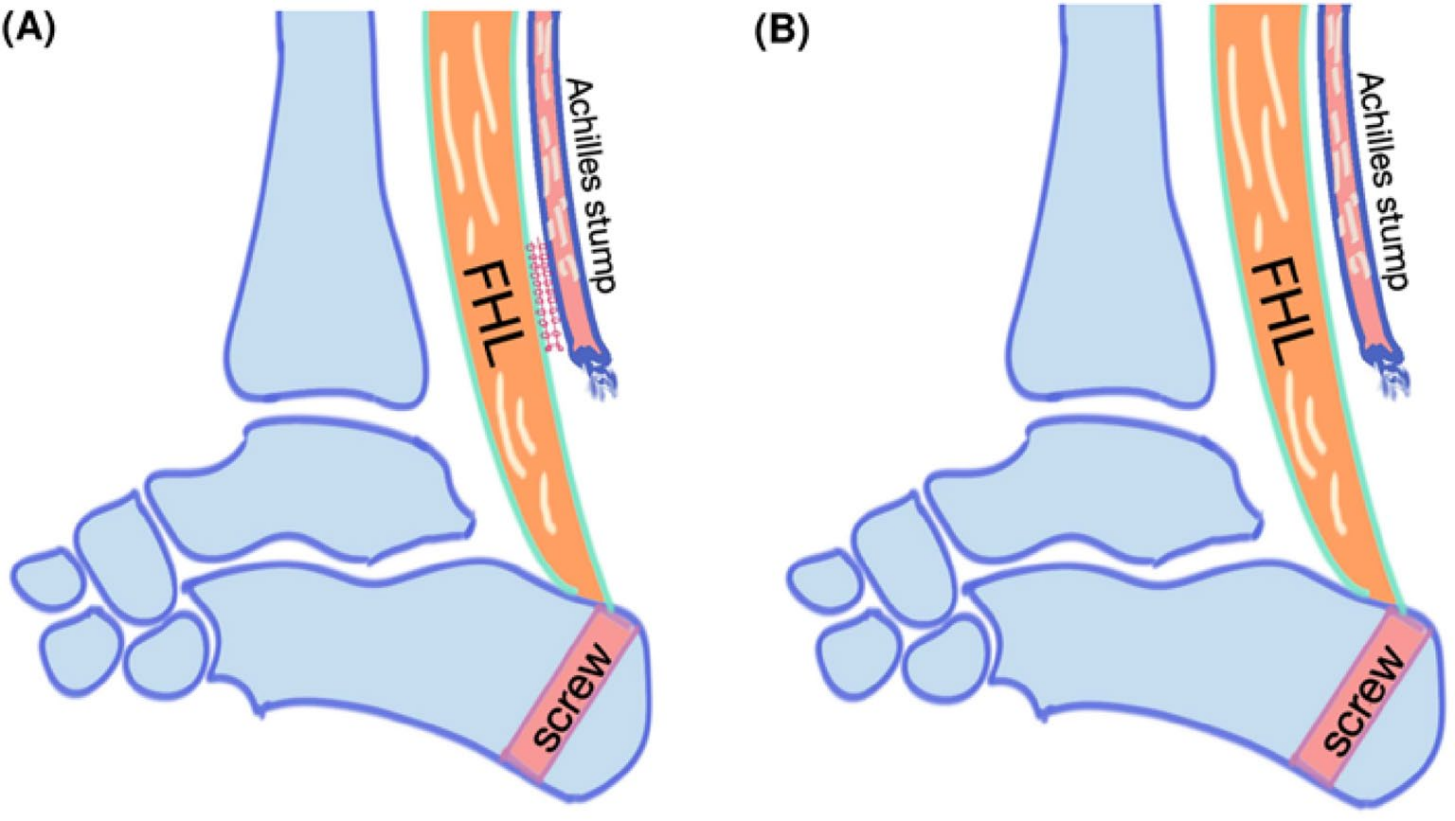
The different managements of the Achilles stump after FHLT (**A**) reattachment of Achilles stump to the transferred FHL side by side (reattachment group) and (**B**) Achilles stumps were left alone without reattachment to FHL (non-reattachment group). All distal part of diseased Achilles stump and its footprint were excised completely in both groups and the FHL was transferred to the prior Achilles insertion on calcaneus with single screw fixation.

Sagittal T1-weighted (T1W), T2-weighted (T2W), and short-tau inversion recovery sequences of the preoperative and postoperative MRI scans were evaluated, and additional axial T1 scans at specific levels were used to compare FHL size differences before and after FHLT. FHL changes and characteristics were as follows: presence of hyper-vascularization (inflammation), homogeneity, and fatty infiltration in the FHL muscle; integration at the reattachment site, union of the transferred FHL to the bone tunnel site; tunnel distance (mm) from the end of the calcaneus; angle of the FHLT tunnel in the sagittal plane; and cross-sectional area of the FHL muscle in the coronal plane. The preoperative cross-sectional area of the FHL muscle was selected and measured in the axial section at 80 mm above the myotendinous junction. However, the myotendinous junction was displaced downward to the entrance of the bone tunnel at the calcaneus after FHLT. The postoperative cross-sectional area of the FHL muscle was measured 80 mm above the bone tunnel of the calcaneus because the FHL tendon had been thoroughly pulled through the tunnel and the new position of the myotendinous junction was at the entrance of the bone tunnel (Fig. 3).

**Figure 4:**
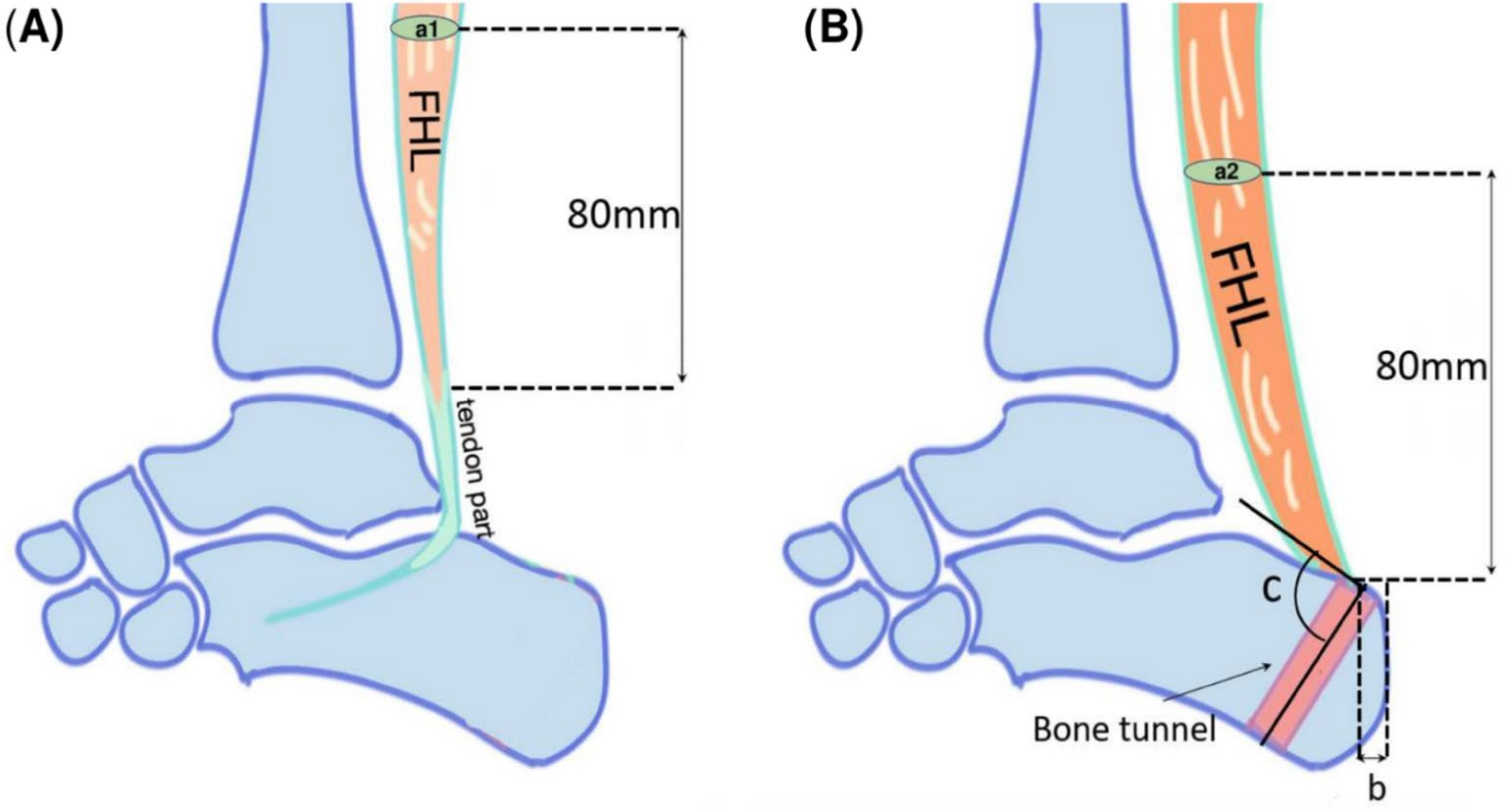
Schema of FHLT and measured parameters. (**A**) Preoperative, (a1) trans-section area of preoperative FHL and (**B**) postoperative, (a2) trans-section area of postoperative FHL, (b) tunnel distance from the posterior tuberosity of the calcaneus to the bottom of bony tunnel, (c) tunnel angle is the angle between the tangential line of the posterior tuberosity calcaneus and bony tunnel.

Fatty infiltration in the FHL muscle was classified according to the Goutallier grading system and Fuchs staging system at the same level before and after surgery, respectively. Goutallier grades, classified as four grades, are defined according to the percentage of atrophy and fatty degeneration of the affected muscle^29^. In the Fuchs staging system, a modified version based on Goutallier classification system, three stages are defined as minimal, moderate and severe according to the intramuscular fat distribution comparing to the muscular mass^30^. The cross-sectional FHL muscle area and the percentage of fatty infiltration in the coronal plane of the MRI section were further calculated using Materialise Mimics Innovation Suite software (ver. 21, Materialise NV, Leuven, Belgium) (Fig. 4).

### Evaluation of outcomes

**Figure 5:**
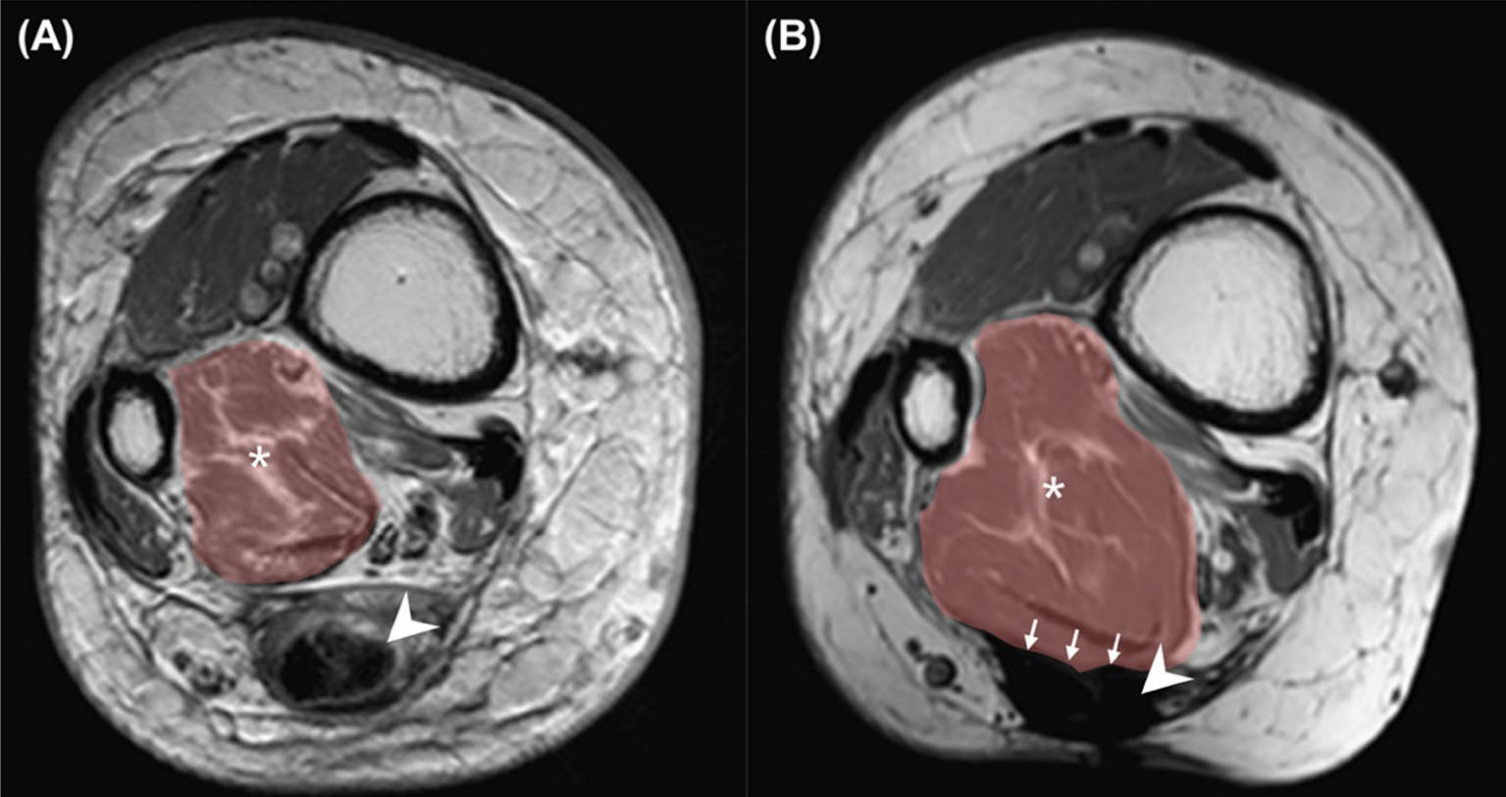
Cross-section of lower limb at the selected level at 80 mm above the myotendinous junction in MRI before and after FHLT from the same case (No. 5, in reattachment group, and the postoperative image was obtained 19 months after index surgery). (**A**) Preoperative and (**B**) postoperative T1W MRI. Cross-sectional area of the FHL muscle belly demonstrated by brown shadow. Asterisk: Fat infiltration in FHL muscle belly area in coronal section. Arrowhead: Achilles tendon. Arrow: the integration between FHL and the reattached site of the Achilles stump.

The assessment of outcomes involved gathering VAS in addition to AOFAS Hindfoot scores both before and after the surgical procedure. Complications were also recorded. All patients were followed up for a minimum of 9 months after FHLT surgery (Fig. 5).

### Statistical analysis

Continuous variables are presented as means and standard deviations or medians and interquartile ranges. Categorical data are presented as frequencies and percentages. Descriptive statistics were used to analyze the parameters of the operated legs before and after the FHLT. Moreover, between-group differences were analyzed. Categorical data were analyzed by chi-square test, and continuous variables were compared between the groups using the Mann–Whitney *U* test or independent t test. The Wilcoxon signed-rank test was performed to examine the differences between the preoperative and postoperative parameters. Spearman correlation coefficient analysis was used to examine the correlation between FHL characteristics and hypertrophy. The cumulative incidences of FHL enlargement over time in both groups were further analyzed using the Kaplan–Meier method and the log-rank test. The probability of FHL enlargement of cases in the two groups during the given follow-up time period was measured and the case numbers at specific time were also recorded on the bottom of Fig. 2. All statistical analyses were performed using SPSS Statistics (ver. 22; IBM Corp., Armonk, NY, USA). The significance level was set at p < 0.05.

### Institutional review board statement

For this study informed consent has been waived by Taichung Veterans General Hospital (CE21051A)/ethics committee due to the anonymity and retrospective nature of the study.

## Data Availability

The datasets used or analyzed during the current study are available from the corresponding author on reasonable request.

## Abbreviations

AOFAS: American Orthopedic Foot and Ankle Society
ASA: American Society of Anesthesiologists
BMI: Body mass index
FHL: Flexor hallucis longus
FHLT: Flexor hallucis longus transfer
IAT: Insertional Achilles tendinopathy
MRI: Magnetic resonance imaging
T1W: T1-weighted
T2W: T2-weighted
USA: United states of America
VAS: Visual analog scale
